# Angular compounding for speckle reduction in optical coherence tomography using geometric image registration algorithm and digital focusing

**DOI:** 10.1038/s41598-020-58454-0

**Published:** 2020-02-05

**Authors:** Jingjing Zhao, Yonatan Winetraub, Edwin Yuan, Warren H. Chan, Sumaira Z. Aasi, Kavita Y. Sarin, Orr Zohar, Adam de la Zerda

**Affiliations:** 1 Department of Structural Biology, Stanford University School of Medicine, Stanford, California 94305 USA; 2 Biophysics Program at Stanford, Stanford, California 94305 USA; 3 Molecular Imaging Program at Stanford, Stanford, California 94305 USA; 4 The Bio-X Program, Stanford, California 94305 USA; 5 Department of Applied Physics, Stanford University, Stanford, California 94305 USA; 6 The Chan Zuckerberg Biohub, San Francisco, California 94158 USA; 7 Department of Dermatology, Stanford University School of Medicine, Stanford, California 94305 USA

**Keywords:** Optical imaging, Imaging and sensing

## Abstract

Optical coherence tomography (OCT) suffers from speckle noise due to the high spatial coherence of the utilized light source, leading to significant reductions in image quality and diagnostic capabilities. In the past, angular compounding techniques have been applied to suppress speckle noise. However, existing image registration methods usually guarantee pure angular compounding only within a relatively small field of view in the focal region, but produce spatial averaging in the other regions, resulting in resolution loss and image blur. This work develops an image registration model to correctly localize the real-space location of every pixel in an OCT image, for all depths. The registered images captured at different angles are fused into a speckle-reduced composite image. Digital focusing, based on the convolution of the complex OCT images and the conjugate of the point spread function (PSF), is studied to further enhance lateral resolution and contrast. As demonstrated by experiments, angular compounding with our improved image registration techniques and digital focusing, can effectively suppress speckle noise, enhance resolution and contrast, and reveal fine structures in *ex-vivo* imaged tissue.

## Introduction

Since its conception^1^, optical coherence tomography (OCT) has proven to be a highly useful and versatile biomedical imaging modality, with the capability of non-invasively acquiring high-resolution, cross-sectional images. It has been widely used for diagnosis in ophthalmology^2,3^, with additional diagnostic capabilities for cancer^4^, cardiology^5^, angiography^6^, dentistry^7^, and dermatology^8^. Moreover, it serves as an important tool for brain and neuroscience research^9,10^, shows potential for generating histology-like images^11,12^, and can work with functionalized contrast agents^13,14^. Like other imaging techniques utilizing spatially coherent waves (including ultrasound), OCT images present with a speckled appearance, making visualization of fine structures difficult, and in some cases, impossible, without speckle reduction. Examples of this include the recognition of the tiny anatomical structures in dermatology, like tactile corpuscles and sweat ducts^15^, identification of different retinal layers^15,16^, and tumor margin delineation in the brain^17^. A common mechanism for speckle reduction is to average images with uncorrelated speckle patterns. Different causes for speckle variation have been studied, including using a wavelength-diverse light source (frequency compounding)^18,19^, spatial compounding of adjacent regions^20^, dynamic speckle illumination (speckle modulation)^15,21^, and illumination from multiple incident angles (angular compounding)^22^. However, the number of speckle patterns generated by frequency compounding is limited by the total bandwidth of the light sources; spatial compounding induces loss of resolution; speckle modulation also causes resolution loss since introducing random phase patterns into the light wavefront makes the illumination area larger and unstable; angular compounding can also produce concurrent spatial compounding and mismatch in the defocused regions because the detection area varies with angle^23^.

At the microscopic level, the essence of speckle variation is to change the phase difference between scatterers. Compared to other speckle reduction methods, angular compounding is capable of continuously and linearly generating changes in phase from 0 to $2\pi$ by a continuous change in incident angle, resulting in a very effective speckle reduction, as analyzed in the discussion section. Feasibility of speckle reduction by angular compounding has been demonstrated^24^. Synchronous multi-angle detection is realizable^25^ at the cost of significant cross talk between angular images. For a large and adjustable angle change, moving the incident beam away from the lens optical axis is a popular method^22,26,27^. When this takes place, the angle-coded optical pathlength and propagation direction make the mapping between real space and image space change with angle. Although some universal image registration algorithms have been applied for angular compounding^16,23,28,29^, none of them are based on geometrical considerations of the beam path, resulting in errors produced in the registration process. The vertical correction^23^ can only guarantee pure angular compounding at the focal plane. The common ‘rotation + translation’ method^28^ does not correct quadratic deformations and can prevent spatial averaging in the focal region within a relatively small scanning range. The pure software method^16,29^ based on similar points in different images may reduce higher-order deformations but requires subjective human input. We build a common geometrical optical model of the angular compounding system. According to it, a geometrically-motivated image registration method is specially designed. It describes the quadratic mapping from the OCT space to physical space and can correct image translation, scaling, rotation, and nonlinear deformation. For angular compounding, it is able to remove inadvertent spatial averaging that is present in existing registration methods, preventing resolution reduction caused by spatial mismatch, and achieve angular compounding in the whole cross-sectional image. Besides the stated application towards correcting angle-coded images, the registration model is also useful for other applications, such as correcting the quadratic deformations of a system in which the scanning mirror is not at the back focal plane of the objective.

Since a focused Gaussian beam is typically used in an OCT system, the transverse resolution in an acquired B-scan image decreases with distance from the focal plane. Digital focusing of OCT images is a promising technique^30–34^ that can solve the trade-off between transverse resolution and the depth-of-field. Digital focusing is developed in this work to additionally complement angular compounding. Its abilities to increase lateral resolution and contrast were demonstrated in the experiments. The idea behind our proposed digital focusing algorithm is the convolution of a complex OCT image and a matched filter. The matched filter is equal to the conjugate of the point spread function (PSF). The PSF is shown to be related to the light distribution function and its expression is derived. This digital focusing method requires no additional hardware additions to the optical system^35,36^ and can operate at different angles.

## Results

### Speckle reduction

**Figure 1:**
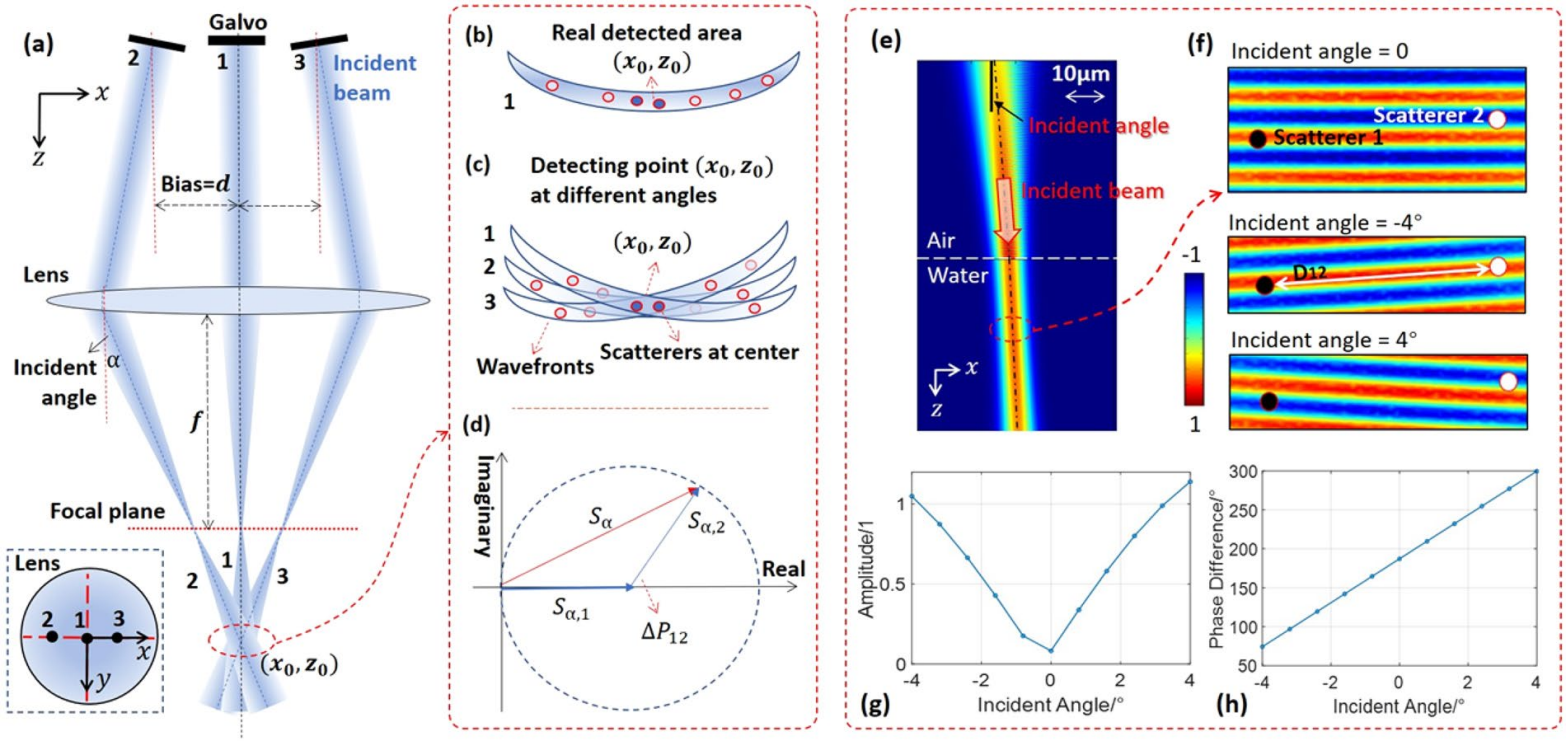
Speckle reduction by angular compounding in an OCT system. (**a**) The incident angle $\alpha$ is manipulated by the offsetting distance $d$, the distance between the lens optical axis and the galvo centerline, and $\alpha \approx \arctan (d/f)$. The scanning direction is along the $x$-direction. (**b**) The detected area for a point is determined by the light wavefronts and the axial resolution. (**c**) The area rotates with the incident angle. The dark blue dots represent strong scatterers and the light blue ones are weak scatterers. (**d**) Speckle can be generated by two strong scatterers with similar scattering amplitudes. (**e**,**f**) Finite element analysis (FEA) simulations by COMSOL software are applied to demonstrate the feasibility of speckle-reduction, the details of which can be found in method section. The light source is 920 nm and the light spatial frequency is doubled in the figure due to the round-trip optical path. The beam is rotated by the incident angle and the optical pathlength difference between the two scatterers is modulated simultaneously, changing the total amplitude (**g**) and phase difference (**h**) of the two scatterers. The distance ${D}_{12}$ is 2 μm.

Angular compounding OCT contains a transversely movable scanner that is responsible for changing the detection angles, allowing one to observe a single point from different angles, as shown in Fig. 1(a). The illumination is performed by a focused Gaussian beam, the expression for which is deduced in Supplementary S1. The detected area representing the point $({x}_{0},{y}_{0})$ is physically determined by the wavefronts (width, the focal size is 8 μm in our OCT system) and OCT axial resolution (height, 2 μm), as illustrated in Fig. 1(b). The gathered signal for the point is the sum of the scattered signals in the whole area, as expressed below

1 $${S}_{\alpha }=\exp (-ik\cdot 2r)\mathop{\sum }\limits_{n=1}^{N}{A}_{n}\exp (-ik\cdot 2\Delta {r}_{\alpha ,n})$$

where $\alpha$ stands for the incident angle, $k$ is the wave number ($=2{\rm{\pi }}/\lambda$, $\lambda$ is the wavelength), $\,r$ is the average optical pathlength, ${A}_{n}$ is the amplitude, $\Delta {r}_{\alpha ,n}$ is the pathlength difference, $(\Delta {r}_{\alpha ,n}+r)$ is the one-way optical pathlength, ${A}_{n}\exp [-2ik(\Delta {r}_{\alpha ,n}+r)]$ is the scattering signal, and $N$ is the number of scatterers. The detected area will rotate with the angle and only the center region can be measured by all the angles, as presented in Fig. 1(c). Without loss of generality, it is assumed that two strong scatters with similar amplitudes are located in the center region. Ignoring the effect of the weak scatterers, the scattered signal is ${S}_{\alpha ,12}=\exp (i{P}_{\alpha ,1}){A}_{1}[1+\exp (i\Delta {P}_{\alpha ,12})]$, where $\Delta {P}_{\alpha ,12}$ is the phase difference equal to $({P}_{\alpha ,2}-{P}_{\alpha ,1})=2k(\Delta {r}_{\alpha ,2}-\Delta {r}_{\alpha ,1})$, and the amplitude is $|{S}_{\alpha ,12}|=\sqrt{2}{A}_{1}\sqrt{1+cos\Delta {P}_{\alpha ,12}}$, as shown in Fig. 1(d). The intensity of the corresponding pixel in the OCT image is proportional to the product of the amplitude $|{S}_{\alpha ,12}|$ and the constant light-amplitude in the reference arm. For simplicity, $|{S}_{\alpha ,12}|$ is used to represent the pixel intensity. The phase difference $\Delta {P}_{\alpha ,12}$ can modulate the intensity from 0 ($\Delta {P}_{\alpha ,12}\in \{sign(m)(2|m|-1)\pi ,\,m=0,\pm \,1,\pm \,2,\cdots \}$) to 2${A}_{1}$ ($\Delta {P}_{\alpha ,12}\in \{2m\pi ,\,m=0,\pm \,1,\pm \,2,\cdots \}$). A zero represents a totally dark pixel and 2${A}_{1}$ represents the brightest one. The distribution of intensities within a speckle pattern is produced in this way.

As one can see, $|{S}_{12}|$ is periodic in the phase difference $\Delta {P}_{12}$ = $(\Delta {P}_{12,0}+\delta O{P}_{12})$ with period $2\pi$, where $\Delta {P}_{12,0}$ is the initial difference and $\delta O{P}_{12}$ is a phase shift. If we can continuously adjust $\delta O{P}_{12}$ from 0 to $\,2m\pi$ $(m=\pm \,1,\pm \,2,\cdots )$, the average pixel intensity will be stabilized at 1.27*A*_1_, as calculated from $(1/2\pi ){\int }_{0}^{2m\pi }|{S}_{\alpha ,12}|d(\delta O{P}_{12})$, which is not affected by the initial phase difference. This is a significant improvement because the original intensity is a random value in the range of 0~2*A*_1_ with a variance of 0.62*A*_1_. This phase average process converts the originally dark and bright pixels to more uniform ones, eliminating speckle noise. Considering that a large phase difference may cause the two scatterers originally contained in one voxel to now be present in two voxels, the phase shift from 0 to $2\pi$ is the best choice. As shown in Fig. 1(e–h), an effective way to produce a phase shift is to change the incident angle, according to the below equation

2 $$\delta O{P}_{12}(\Delta \alpha )=\Delta {P}_{\alpha +\Delta \alpha ,12}-\Delta {P}_{\alpha ,12}\approx 2(k\cdot RI){D}_{12}\Delta \alpha $$

where *D*_12_ is the physical distance between the two scatterers, $\Delta \alpha$ is the angle change, and $RI$ is the refractive index. It is desirable for the achievable upper limit of the phase shift to be $2\pi$, meaning that ${D}_{12}\Delta \alpha$ is no less than a half wavelength. In this study, limited by the lens aperture (LSM03-BB, Thorlabs), the largest angle change is 8°. And the light source center wavelength is 920 nm (Ganymede SD-OCT, Thorlabs). Thus, the lower limit of ${D}_{12}$ is 3.3 μm, which is smaller than the 8 μm focus size (1/e^2^). If using the radius of focal spot ${R}_{S}$ as the lower limit, we can plug $\Delta \alpha \approx \Delta d/(f\cdot RI)$ and ${R}_{S}=2\lambda f/\pi {D}_{B}$ (${D}_{B}$ is the beam diameter, 5.4 mm in our system) into Eq. (2) and get $\Delta {d}_{max}=0.8{D}_{B}$. In experiments, 11 angles, [−4°,−3.2°, −2.4°, −1.6°, −0.8°, 0°, 0.8°, 1.6°, 2.4°, 3.2°, 4°], are applied and the movement range of $d$ is from −2.5 mm to 2.5 mm.

### Image registration

Without correct image registration, the speckle reduction capability of angular compounding cannot be appreciated. The image registration model seeks to find the real-space position $(x,z)$ of every pixel $(u,v)$ in a single OCT B-scan images taken at different angles. The expression is given in Supplementary S2 and S4 and can be simply expressed as $x={f}_{x}(u,v,d,{u}_{f},{v}_{f})$ and $z={f}_{z}(u,v,d,{u}_{f},\,{v}_{f})$, where $({u}_{f},{v}_{f})$ refers to the focus, and *d* is the bias distance for changing the incident angle as shown in Fig. 1(a). The focus position is basically independent of the incident angle and can work as a reference point. By fusing the angular images after the correction of image registration, the composite image is produced, and its quality can be evaluated by the ratio

3 $${R}_{HF}=\int {\int }_{HF}|F{T}_{x,z}[CI(x,z,{u}_{f},\,{v}_{f})]|/\,\,\int {\int }_{All}|F{T}_{x,z}[CI(x,z,{u}_{f},\,{v}_{f})]|$$

where $F{T}_{x,z}$ stands for Fourier transform along *x*- and *z*-directions, $CI$ is the composite image, $HF$ means high frequencies of the Fourier transform, $All$ represents the total frequencies, ${R}_{HF}$ is the ratio of high frequencies to the all. The average size of structures in the composite image will reach the minimum when the image registration achieves its best performance, resulting in the maximum ratio. In practice, $F{T}_{x,z}$ is calculated from built-in Matlab functions, $F{T}_{x,z}=|fftshift(fft2(CI))|$, here $fft2$ is for the two-dimensional (2D) fast Fourier transform and $fftshift$ shifts the zero-frequency component to the center of the matrix. The $HF$ region is given by removing a small central area containing the low frequencies from the $F{T}_{x,z}$ matrix. In our cases, the width of the central area is chosen as one to three hundredths of the width of $F{T}_{x,z}$, and the height is four to six hundredths that of $F{T}_{x,z}$.

### Digital focusing

According to the principle of synthetic aperture optics, digital focusing is developed in order to improve the lateral resolution and contrast. Every B-scan image can be processed by the digital focusing algorithm before image registration. As shown in Supplementary S1, the light distribution for detection is expressed as ${U}_{L}=A(x,z,\theta ,d)\exp [ik\cdot r(x,z,\theta ,d)]$, where $A$ is the amplitude, $k$ is wave number, $r$ is the optical pathlength, and $\theta$ is the scanning angle of the galvo. According to the analysis in Supplementary S3 and the ref. ^30^, the point spread function (PSF) is approximately $PSF=A(u,v,\theta ,0)\exp [i2k\cdot r(u,v,\theta ,0)]$. The optical pathlength is two times $r$, since only the back-scattered light that propagates in the same direction as the incident light can be collected due to the confocal gate. The matched filter is designed as the conjugate of PSF, which equals to $MF=A(u,v,\theta ,0)\exp [-2k\cdot r(u,v,\theta ,0)]$. To normalize the vertical amplitude, $MF$ is multiplied by $1/{{\rm{\max }}}_{v}(A)$, where ${{\rm{\max }}}_{v}(A)$ means the maximum value at the depth $v$. The digitally focused image is generated by the convolution of the two-dimensional filter $MF$ with every complex B-scan image, along the scanning direction $u$. Considering the focus movement caused by the sample-air interface^37^, the matched filter should be finely adjusted vertically to achieve good performance according to the criterion proposed by Eq. (3). Since digital focusing seeks to enhance the horizontal resolution, the Fourier transform is operated only in the horizontal direction, and the ratio is ${R}_{HF}=\int {\int }_{HF}F{T}_{x}(DF)/\int {\int }_{All}F{T}_{x}(DF)$, where $DF$ is the digitally-focused image.

### Agar-bead phantom

As a sparse sample, the phantom composed of agar gel (J.T.Baker 500G-Agarose) and 1 μm Polystyrene (PS) beads (3.64 × 10^7^ beads/ml, Spherotech) is able to clearly demonstrate the capabilities of angular compounding and digital focusing. The phantom was observed at eleven angles from −4° to 4°, and 20 B-scans were taken for a single angle in order to remove photon noise, with 220 B-scans captured in total. To mimic a normal OCT image capture, the phantom was imaged with vertical illumination for 11 times (11 × 20 B-scans). The scanning step is 0.976 μm. Images are displayed on a logarithmic scale. This sampling strategy was also applied to the rest of the experiments in this work.

**Figure 2:**
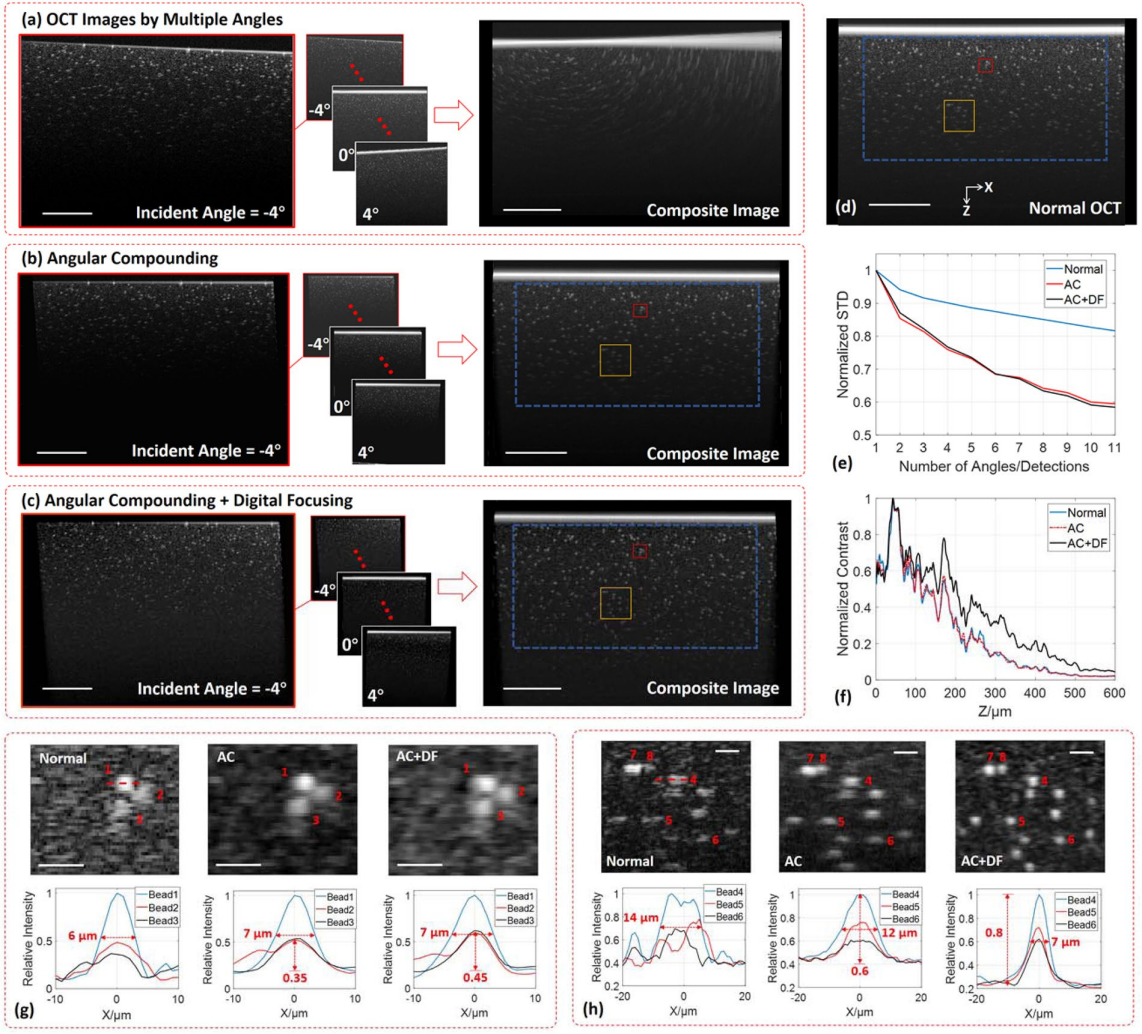
Demonstrations of angular compounding and digital focusing by using the agar phantom containing 1 μm PS beads. (**a**) Eleven angles, [−4.0°, −3.2°, −2.4°, −1.6°, −0.8°, 0°, 0.8°, 1.6°, 2.4°, 3.2°, 4.0°], whilst imaging the same area, and the composite image created by directly overlapping the 11 angular OCT images is entirely mismatched. Twenty B-scans are taken for every angle to reduce random noises. (**b**) The angular compounded image is produced by fusing the correctly registered angular images. (**c**) With digital focusing, the composite image presents better horizontal resolution and deeper penetration. (**d**) The image composed of 220 B-scans (20 × 11) by a normal OCT system is used as a control group. (**e**) The reduction in the background standard deviation (STD) versus the number of angles for AC and AC + DF. In the normal OCT, the data is captured at zero angle 11 times. The regions for STD calculations are marked by the blue rectangles in (**b–d**). (**f**) The contrast varies with depth, and Z is the vertical distance to the sample surface. (**g**) The zoom-in views of the red rectangles in (**b–d**) show three adjacent beads close to the focal plane and their intensity profiles along the horizontal direction (on the linear scale). (**h**) The zoom-in views of the yellow rectangles in (**b–d**) show several beads away from the focal plane. AC, angular compounding; DF, digital focusing; STD, standard deviation. Scale bar, 200 μm in (**a–d**), 50 μm in (**g**,**h**).

As shown in Fig. 2(a), the OCT images by different incident angles are coded by angle, meaning that the location of a bead in each image changes with the angle, and directly overlapping these images will form a mismatched image where the beads exhibit apparent artifacts. The corrected angular compounding (AC) is achieved by our image registration model that map the pixels in every angular image to their real-space positions. The registered images are fused into the composite image, as illustrated in Fig. 2(b), in which the bead profiles are clear and symmetrical. Furthermore, nearly constant lateral resolution and deeper penetration are realized by digital focusing (DF), as demonstrated in Fig. 2(c). Supplementary S4 compares this image registration method with the common ‘rotation + translation’ method^26^. The image taken by normal OCT acquisition is shown in Fig. 2(d). Comparing the normalized standard deviation (STD)^15^ of the background (excluding the beads based on the intensity difference, as at one depth, the weakest intensity of the top 10% brightest pixels is used as the threshold value for the beads and background) in the regions marked by the blue dashed boxes in Fig. 2(b–d), the reduction in STD by angular compounding is significantly larger than that by normal OCT acquisition, as shown in Fig. 2(e). This effect takes place because simply increasing the number of B-scans only eliminates random noise but has no effect on speckle noise, which is due to the optical path differences between scatterers. Since digital focusing does not physically change the optical path differences either, the angular compounding images with or without digital focusing present almost the same trend in Fig. 2(e). Digital focusing enhances the contrast (contrast = (bead intensity) ÷ (background intensity) − 1) and deepens the penetration within the image, as shown in Fig. 2(f). The penetration depth is improved from 400 μm to 500 μm. In addition, the contrast-to-noise ratio (CNR) of the images generated by normal OCT or angular compounding are around 10–15 dB within the depth of 400 μm, but with digital focusing, the CNR is maintained at 12–18 dB within a 500 μm depth range.

Marked by red and yellow boxes, two examples are selected from Fig. 2(b–d), and their zoom-in views are shown in Fig. 2(g,h). The first example is close to the focal plane and contains three neighboring beads. As illustrated in Fig. 2(g), the speckle noise in the normal OCT image is so strong that it is difficult to recognize Bead 2 and Bead 3, while this is not a problem for angular compounding since we can see their clear profiles in both of ‘AC’ and ‘AC + DF’ images. Besides the direct visual evidence, the relative STD (=STD/Mean) of the image background is calculated to evaluate the speckle intensity, which is 0.17, 0.08, and 0.11 in ‘normal’, ‘AC’, and ‘AC + DF’ images. Observing the cross sections through the three beads (intensity profile along the red dashed line), shows that angular compounding causes the profiles of Bead 2 and Bead 3 to be more Gaussian. With digital focusing, the intensities of Bead 2 and Bead 3 increase by 28% (=0.45/0.35 = 1.28). The bead diameter is 7 μm (full width at half maximum, FWHM), which is a reasonable value since it is close to the lens resolution (6.8 μm, FWHM). The second example is beneath the focal plane and consists of multiple beads, as shown in Fig. 2(h). The beads are masked by speckles in normal OCT, leading to fragmentary profiles. By suppressing speckle, angular compounding can depict the complete bead profiles. Furthermore, digital focusing converts the bead profiles from ellipses into circles. Benefitting from the better resolution, the diameter of Bead 4 improves from 12 μm to 7 μm, and Bead 7 and Bead 8 can be resolved in the ‘AC + DF’ image. Thanks to the contrast enhancement, in the comparison between ‘AC’ profile and ‘AC + DF’ profile, the contrast of Bead 4 intensity to the background grows from 1.5 to 3.5, and CNR increases from 13.5 dB to 15.3 dB.

### Onion

**Figure 3:**
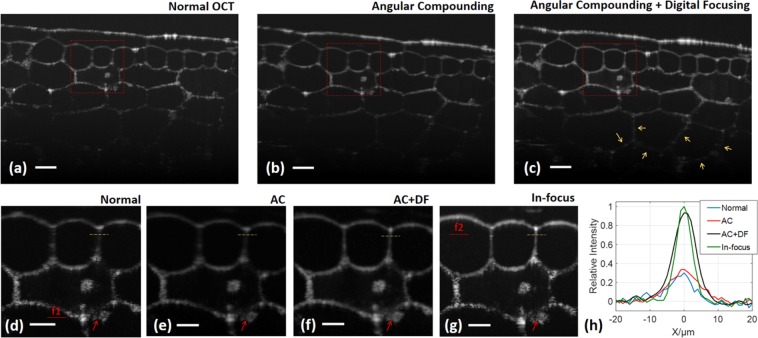
Speckle reduction and contrast enhancement in an onion sample. (**a–c**) The sample is respectively captured/processed by normal OCT, AC, and AC + DF. (**a–f**) The close-up views of the red rectangles in (**a–c**). Line f1 in (**d**) locates the focal plane for (**a–f**). (**g**) In normal OCT, the focal plane is moved to line f2 to take an in-focus image of the cell wall marked by the yellow dashed line. (**h**) The intensity profiles along the yellow lines in (**d–g**) are normalized to the maximum of all four curves, which is the peak of the ‘in-focus’ profile in this case. AC, angular compounding; DF, digital focusing. Scale bar, 100 μm in (**a–c**), 50 μm in (**d–g**).

Featuring a simple and regular structure of elongated cells^38^, onion epidermal tissue is an ideal sample to evaluate the performance of angular compounding. Images given by normal OCT, angular compounding, and angular compounding with digital focusing are shown in Fig. 3(a–c). Visually, digital focusing provides better visibility of the deep structures (cell walls indicated by yellow arrows) in Fig. 3(c). Figure 3(d–f) present the close-up views of the marked regions. In Fig. 3(d–f), strong speckle exists on the cell walls in normal OCT but are diminished with angular compounding, and the tiny structure indicated by the red arrow becomes clear in the ‘AC’ and ‘AC + DF’ images. The red line f1 in Fig. 3(d) is the focal plane for Fig. 3(a–f). Cell walls in the upper halves of Fig. 3(d,e) are blurred due to defocusing, while they are refocused by digital focusing in Fig. 3(f). In Fig. 3(g), the focal plane is moved to line f2 in order to take an in-focus image of the area around the yellow dashed line. The lateral intensities of the cell wall along the yellow dashed lines in Fig. 3(d–g) are plotted in Fig. 3(h). The ‘AC’ profile is smoother than the ‘normal’ one, and the ‘AC + DF’ profile is pretty close to the profile achieved by the ‘in-focus’ image. The CNRs for the four profiles are respectively 7 dB, 11 dB, 16 dB, and 14 dB, and the cell wall sizes (FWHM) given by ‘AC’, ‘AC + DF’, and ‘in-focus’ are 10 μm, 7 μm, and 5.5 μm.

### Human skin

The primary application of OCT is in the area of biomedical imaging. Two samples of fresh human skin were processed by angular compounding (AC) OCT with digital focusing (DF). The improvements compared to normal OCT are analyzed below.

**Figure 4:**
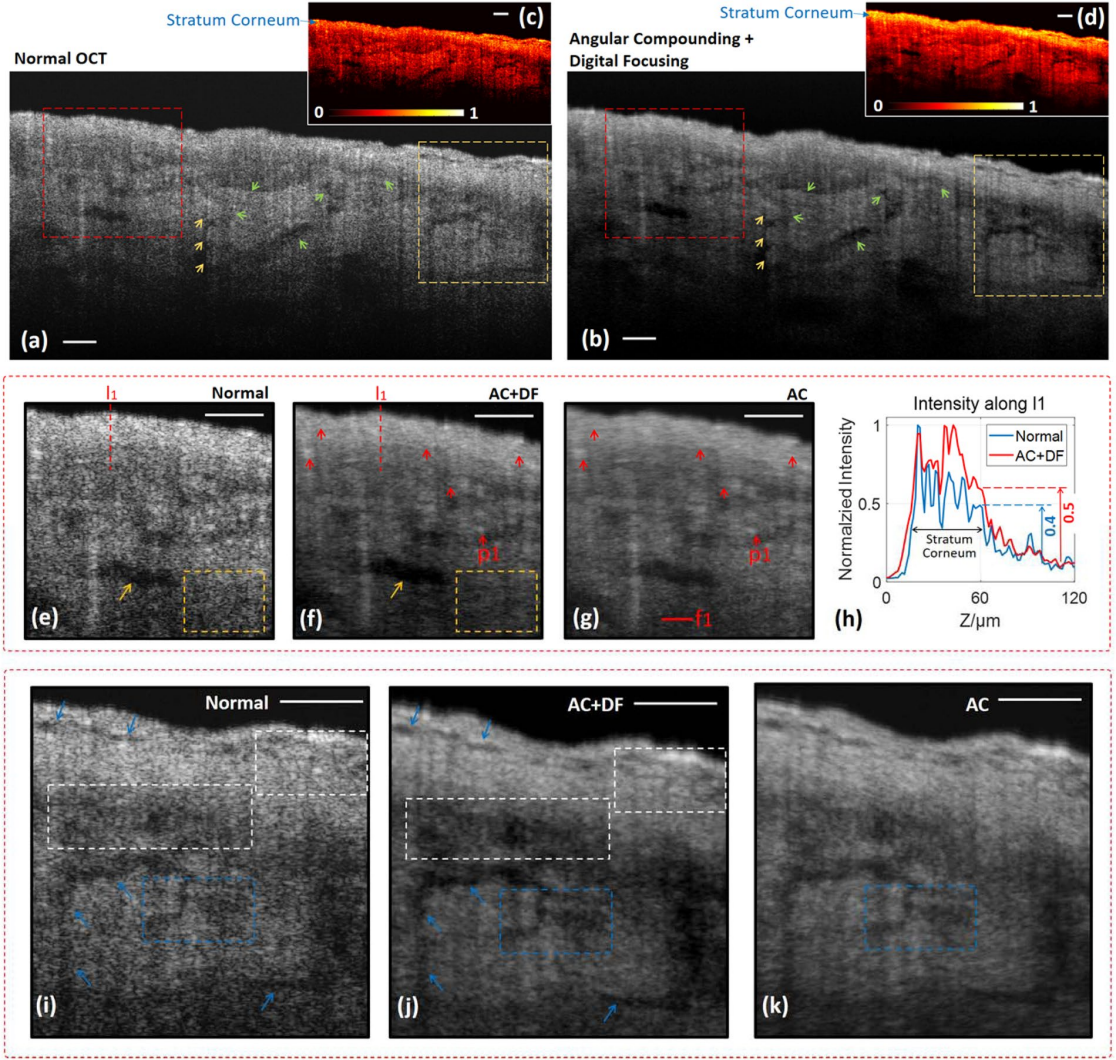
Human skin at shin (89 years, female). The images yielded by normal OCT (**a**) and angular compounding with digital focusing (**b**), the zoom-in views of the red and yellow rectangles are shown in (**e–g**) and (**i–k**). (**c,d**) The gray images are color-coded by using the ‘hot’ colormap. The focal plane is marked by the red line f1 in (**g**). (**h**) The intensity profile along line l1, which is the average of 20 horizontally adjacent lines. The epidermis-dermis interface in the ‘AC + DF’ image is shaper with better contrast. AC, angular compounding; DF, digital focusing. Scale bar, 100 μm.

The first sample is skin from the shin of an 89-year-old female In Fig. 4(a,b), speckle reduction allows visualization of the clear boundaries of the anatomical structures, such as the profiles of the three semi-circular features (yellow arrows) and the interfaces between structures (green arrows). The zoom-in views of the two selected regions are respectively shown in Fig. 4(e–g,i–k). Speckle suppression by our techniques makes the boundary of the hole (orange arrow) shaper and clearer in Fig. 4(e,f), deblurs the contents of the orange box in Fig. 4(e,f), enhances the visibility and contrast of the crack-like structures (blue arrows and blue rectangle) and the tiny structures (white boxes) in Fig. 4(i,j). Other similar examples can be found by further examining the images. Digital focusing works for this densely scattering sample. In terms of resolution, as demonstrated in Fig. 4(f,g), the fine structural details pointed out by the red arrows are spatially resolved more clearly, for example, the horizontal size of point p1 is 14 μm (FWHM) in ‘AC’ image and 10 μm in ‘AC + DF’ image., The image processed by digital focusing shows superior contrast. The maximum intensity difference that blue rectangle in Fig. 4(j) achieves is 11.5 dB, while in Fig. 4(k) it is 10.3 dB. Further evidence for the image quality improvement is that the epidermis layer becomes more identifiable in the ‘AC + DF’ images, as shown in Fig. 4(c,d),(h).

**Figure 5:**
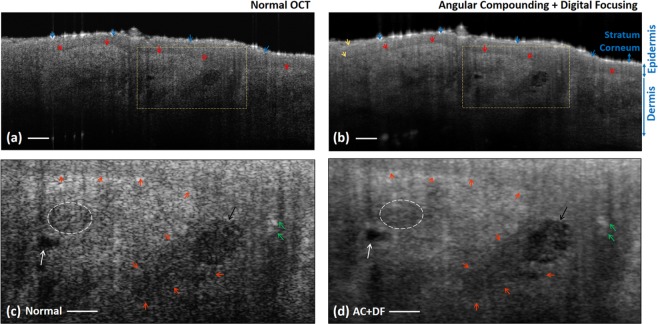
Speckle reduction and increased visibility in human cheek skin (60 years, male). The images are taken by normal OCT (**a**) and AC + DF (**b**), and the zoom-in views of the marked regions are shown in (**c,d**). AC, angular compounding; DF, digital focusing. Scale bar, 100 μm.

The second sample is skin from cheek (60 years, male). In Fig. 5(a,b), although the stratum corneum boundary (blue arrows) and the epidermis-dermis interface (red arrows) are distinguishable in normal OCT, they look more continuous and have higher contrast in the ‘AC + DF’ image, especially for the left-most segment indicated by the yellow arrows. Figure 5(c,d) are the zoom-in images of the marked areas. Once again, the visibility improvements in the ‘AC + DF’ image are demonstrated by the clearer epidermis-dermis interface (orange arrows), the sharper profile of the vessel-like structure (white arrow), the recovered shape of the two semi-circular features (green arrows), and the newly revealed tiny structures in the hole-like structure (black arrow). Moreover, in Fig. 5(c,d) the maximum intensity difference of ‘AC + DF’ image is 4.2 dB larger than that of the ‘normal OCT’ image.

## Discussion

Without the correct registration of the images taken at different angles, pure angular compounding is not achievable for all depths due to defocusing in the composite image. The introduced spatial compounding contributes to resolution reduction and image blurring in the off-focal regions. The image registration model developed in this work solves this problem by calculating the real-space location of a pixel from its angle-coded coordinates in the OCT image, making the speckle suppression by pure angular compounding available for all depths. Limited by the nature of the Gaussian beam, lateral resolution decreases in the off-focal planes. Therefore, digital focusing is implemented to complement angular compounding, for generating greater resolving power and higher contrast.

Here the principle of speckle reduction is further analyzed. Equation (1) reveals that the phase differences between scatterers in one voxel can modulate the scattering intensity between the darkest and the brightest intensities. The average of the intensities produced by the phase differences from 0 to 2π approaches a stable value that is dominated by the scattering amplitudes of the individual scatterers, and the speckle is removed by using this average value to represent the voxel intensity. This ergodic process reduces the coherence between scatterers, which is approximatively expressed as

4 $$|S|=|{\sum }_{n=1}^{N}{A}_{n}\exp (-i\cdot 2\Delta {P}_{1,n})|\to {\left(\frac{1}{2\pi }\right)}^{N}\left({\int }_{0}^{2\pi }\cdots {\int }_{0}^{2\pi }|S|d\Delta {P}_{1,1}\cdots d\Delta {P}_{1,N}\right)\approx f\left({A}_{1},{A}_{2},\cdots ,{A}_{n}\right)$$

where $S$ stands for the scattering intensity, ${A}_{n}$ is the amplitude of one scatterer, $\Delta {P}_{1,n}$ is the phase difference between the $n\,$th scatterer and the first one, and $f$ represents a function of scattering amplitudes. In this work, the modulation of the phase difference is realized by using multiple incident angles. Other methods for adjusting phase differences also have the potential to diminish speckle. For example, one can directly rotate the sample with small angles^39^ or artificially cause tiny deformations inside the sample^40^. If the scattering amplitudes of the scatterers in one voxel can be changed with different variations, the averaging strategy will be conducive to speckle reduction, for example, as is the case with random speckle illumination^15,41^. Apparently, using several light sources with different center wavelengths is another method because the phase difference is inversely proportional to wavelength^42^. Among the above methods, changing incident angle is optimal because of its easy realization of the $2\pi$ change in phase difference for every pair of scatterers. The situation is complex when one voxel has multiple strong scatterers. According to Eqs. (2) and (4), the signal scattering from one voxel is written as

5 $$|S(\Delta \alpha )|=|{\sum }_{n=1}^{N}{A}_{n}\exp (-i\cdot 2\Delta {P}_{1,n})exp\left(-i\cdot 2k{D}_{1,n}\Delta \alpha \right)|\to \frac{1}{\tau }\left({\int }_{0}^{\tau }|S|d\Delta \alpha \right)\approx f\left({A}_{1},{A}_{2},\cdots ,{A}_{n}\right)$$

where ${D}_{1,n}$ is the physical distance between the $n\,$th scatterer and the first one (its sign is positive if a positive angle change can decrease the phase difference), the period for every item is $(\lambda /2{D}_{1,n})$, and *τ* is the least common multiple of these periods in order to cover all the possible cases, and can be a large value. It indicates that the more scatterers in one voxel, the larger the maximum angle change is required. On the other side, a greater number of scatterers will endow a new incident angle with a higher likelihood of breaking the conditions for generating speckle. In this context, although angular compounding with a relatively small range of angles cannot average all the possible intensities of the voxel containing multiple scatterers, it is still able to effectively reduce speckles.

The proposed image registration model can describe the quadratic mapping from the OCT space to the physical space. Although the model is based on the explicit geometrical structure of the optical system, the analysis in Supplementary S4 proves that an easy calibration procedure can simplify the model and make it equivalent to the simpler ‘rotation + translation’ model. The digital focusing algorithm developed here is based on the convolution of the complex OCT image and the 2D matched filter (MF), which is the conjugate of the point spread function (PSF). The algorithm can be expressed as $con{v}_{u}(MF,OCT)=F{T}_{u}^{-1}[F{T}_{u}(MF)\cdot F{T}_{u}(OCT)]$, where $con{v}_{u}$ and $F{T}_{u}$ present the convolution and Fourier transform along the horizontal direction $u$, respectively. Compared to the traditional digital focusing methods that only involve the phases, the operator in the spatial frequency domain, $F{T}_{u}(MF)$, has a Gaussian envelope in amplitude and works like a low-pass filter. Due to this, the horizontal resolution by $MF$ is a little larger than that given by the matched filter with unity magnitude (uniform amplitude profile in frequency domain), $M{F}_{U}=F{T}_{u}^{-1}\{exp[i\cdot Phase(F{T}_{u}(MF))]\}$, where $Phase$ function returns the phase angle for each element of $F{T}_{u}(MF)$. In our case, the disadvantages of $M{F}_{U}$ include the reductions in penetration depth and contrast, as shown in Supplementary S5. For these reasons, $MF$ is used for our experiments. One limitation of the digital focusing algorithm in this work is that it utilizes the data acquired from a single $xz$ plane and thus, strictly speaking, PSF is only valid for the scatterers in the $xz$ plane. Resultantly, this defocus correction method cannot fully correct the defocus contributed by out-of-plane scatterers. The more accurate re-focused image can be generated when both the $x$ and $y$ spatial frequencies are taken into computation, and 3D scanning is necessary^34^. The light source may affect the digital focusing. Generally, the swept source can offer a quicker scanning speed with balanced photodetectors instead of a spectrometer, but suffers from worse phase stability due to the moveable elements inside^33,43,44^, and the supercontinuum source can reach higher fluence with a wider bandwidth but it is nosier and worse in phase stability^45,46^. The superluminescent diode source is more stable in phase, beneficial to digital focusing^43,47^, which is used in our system. We did not test our methods with a swept-source or a supercontinuum source. Theoretically, the geometrical image registration is not influenced by the type of light source, while the performance of digital focusing is related to the phase stability of the light source.

The main challenge faced by the current implementation of angular compounding is that non-synchronized detection and mechanical movements for individual angles are required, resulting in a much longer total sampling time than with a normal OCT, prohibiting real-time imaging. One potential solution is to establish multiple optical channels for single angles using the same light source^48^. Based on this idea, a design is proposed in Supplementary S6. During one B-scan acquisition, all the points of the sample will be illuminated at these pre-set angles and the collected data can be processed with image registration concurrently, giving a speckle-removed image in real time. Digital focusing can also be performed simultaneously by the software. Furthermore, this design contains no movable parts, which is beneficial to the system robustness and stability. Another interesting research question for multi-angle detection in OCT is to study the three-dimensional (3D) angles of the incident beams. In this work, the optical paths of the illumination lights are contained in the $xz$ plane, as shown in Fig. 1(a). We expect that observing at 3D angles can not only improve image quality and accuracy but also reveal new information, as demonstrated by the measurement of Henle’s fiber layer in the eye^49^.

## Method

### Experimental setup

The OCT system is based on a commercial spectral-domain instrument (Ganymede with a user-customizable scanner OCTP-900, Thorlabs). The central wavelength of the superluminescent diode is 920 nm with a bandwidth of 200 nm, offering an axial resolution of 2 μm (in the air), and the diameter of the single-mode beam is 5.4 mm. The lens LK3-BB used to focus the beam has a focal length of 36 mm and provides a lateral resolution of 8 μm (1/e^2^, or 6.8 μm for FWHM). The galvomirror-objective distance is about 59 mm. Although it is optimal to place the galvanometer mirror in the back focal plane of the lens, it is not achievable in our setup since the lens has to be installed on a separate and static mount (not directly on the scanner) for the horizontal movements of the scanner and an additional space is required between the galvomirror and the lens. The adverse effects introduced are analyzed in Supplementary S4. For detection, the lens and the sample are fixed, and the scanner is horizontally moved (along the scanning direction) by a linear motor (Z812, Thorlabs) to adjust the offset distance between the lens axis and the scanner’s optical axis. Corresponding to the 11 incident angles, [−4.0°, −3.2°, −2.4°, −1.6°, −0.8°, 0°, 0.8°, 1.6°, 2.4°, 3.2°, 4.0°], 11 offset distances are implemented, [−2.5 mm, −2.0 mm, …, 0, …, 2 mm, 2.5 mm]. The 2048-pixel spectrometer acquires A-scans at the rate of 30 kHz, the highest rate accessible in the system. A high acquisition rate will reduce the potential phase drifts of the light source. The scanning step is 0.976 μm. For single detection, 20 B-scans are acquired in order to reduce random noises by averaging. The coordinate system of the lens is calibrated to the coordinate system of the scanner by using the method developed in our previous work^28^. The sample refractive index is 1.34 for agar gel^50^, 1.34 for onion^51^, and 1.41 for human skin^52^.

### Human samples

Human tissue specimens that would otherwise have been discarded during surgical excision of skin growths were collected, placed in keratinocyte media, and stored at 4 °C for an average of four hours before being transported via a courier service to the laboratory. The specimens were stored again at 4 °C in the laboratory refrigerator and would be imaged within six hours after being embedded in agar gel in Petri dishes. Informed consent was obtained from all subjects. All experimental protocols were approved by the Stanford Institutional Review Board (Protocol #48409), and all methods were carried out in accordance with relevant guidelines and regulations.

### Finite element analysis

The 2D FEA model is built using the ‘electromagnetic waves, frequency domain’ module of COMSOL software. It consists of two materials, air in the upper half space and water in the lower half space, as shown in Fig. 1(e). The ‘scattering boundary condition’ is used for the top boundary of the whole rectangular space, where the incident wave enters into the calculation domain, and the other three boundaries are ‘perfect electric conductor’. The incident wave on the top boundary is given by the electric field of ${A}_{I}$ and the normal direction of $(\partial {P}_{I}/\partial x,\,\partial {P}_{I}/\partial y)$, where ${A}_{I}$ and ${P}_{I}$ are the beam amplitude and phase, respectively. The incident Gaussian beam is expressed as ${A}_{I}\exp (i{P}_{I})=[({w}_{0}/{w}_{b})\exp (-{x^{\prime} }^{2}/{w}_{b}^{2})]\exp \{-iky^{\prime} -ik{x^{\prime} }^{2}/[2y^{\prime} +2{R}_{y}^{2}/y^{\prime} ]+i\arctan (y^{\prime} /{R}_{y})\}$, where $k$ is the wave number, $\,{w}_{0}$ is the waist radius, ${w}_{b}$ represents ${w}_{0}\cdot {\rm{sqrt}}[1+({y^{\prime} }^{2}/{R}_{y}^{2})]$, ${R}_{y}$ is the Rayleigh range of $\pi {w}_{0}^{2}/\lambda$, ($x^{\prime} =(x-{x}_{0})\cos \,\alpha -(y-{y}_{0})\sin \,\alpha$, $y^{\prime} =(x-{x}_{0})\sin \,\alpha +(y-{y}_{0})\cos \,\alpha$) are the rotated coordinates, $\alpha$ is the incident angle, and $({x}_{0},{y}_{0})$ is the center of the focus predicted by the propagation directions of the incident lights. Triangular meshes are generated to subdivide the FEA model into discrete geometric cells with the size limitation of no more than one-eighth wavelength. Eleven incident angles are simulated. In each case, the complex amplitudes of the two points, as illuminated in Fig. 1(f), are used for the calculations of the absolute value of the sum of the two complex numbers, Fig. 1(g), and the phase difference, Fig. 1(h).

### Data process flow

First, we scan the sample through 11 angles, 20 B-scans for each angle. Then, the data is processed by the code as follows.

1. PREPARATION. Convert the spectral interferences to spatial information via the Fourier transform, yielding the raw OCT complex B-scan images. Calculate an angular image by averaging the absolute values of its 20 B-scan images, 11 angular images in total.
2. FOCUS. Recognize the sample surface in each angular image by observing intensity differences. Estimate the focus position by locating the pixel that has nearly equal distances to the sample surfaces in all the different angular images or the smallest possible average distance. According to Eq. (3), find the accurate position of the focus.
3. DIGITAL FOCUSING. Apply the proposed digital focusing to process every complex B-scan image, then calculate the new angular images by averaging the absolute image values.
4. IMAGE REGISTRATION. Register the 11 digitally-focused angular images from OCT space to physical space, and combine them to be one fused image.

For the data of 11 angles × 20 B-scans × (1024 × 1024 pixels), the computing cost for the above four steps is about 20 mins (by Matlab, CPU i7-8700, 64 g RAM). Sometimes, tiny bulk movements (a few pixels) of the sample in the vertical direction may occur due to environmental vibrations. A test program can be added after Step 2, which is able to detect such small movements by comparing the surface locations in the registered images and further vertically translate the raw OCT images for offset.

## Supplementary information

supplementary material.
