# Supplementary material for: Angular compounding for speckle reduction in optical coherence tomography using geometric image registration algorithm and digital focusing
Source: Sci Rep. 2020 Feb 5;10:1893. doi: 10.1038/s41598-020-58454-0 (PMC7002526; doi:10.1038/s41598-020-58454-0)
Supplement: Supplementary file 1 — supplementary material. [file 41598_2020_58454_MOESM1_ESM.pdf]

**Angular compounding for speckle reduction in optical coherence tomography using geometric image registration algorithm and digital focusing**

**Jingjing Zhao[1], Yonatan Winetraub[1,2,3,4], Edwin Yuan[5], Warren H. Chan[7], Sumaira Z. Aasi[7], Kavita Y. Sarin[7], Orr Zohar[1], *Adam de la Zerda[1,2,3,4,6]**

[1]Department of Structural Biology, Stanford University School of Medicine, Stanford, California 94305, USA

[2]Biophysics Program at Stanford, Stanford, California 94305, USA

[3]Molecular Imaging Program at Stanford, Stanford, California 94305, USA

[4]The Bio-X Program, Stanford, California 94305, USA

[5]Department of Applied Physics, Stanford University, Stanford, California 94305, USA

[6]The Chan Zuckerberg Biohub, San Francisco, California 94158, USA

[7]Department of Dermatology, Stanford University School of Medicine, Stanford, California 94305, USA

*Correspondence to: adlz@stanford.edu

**S1: Beam profile**

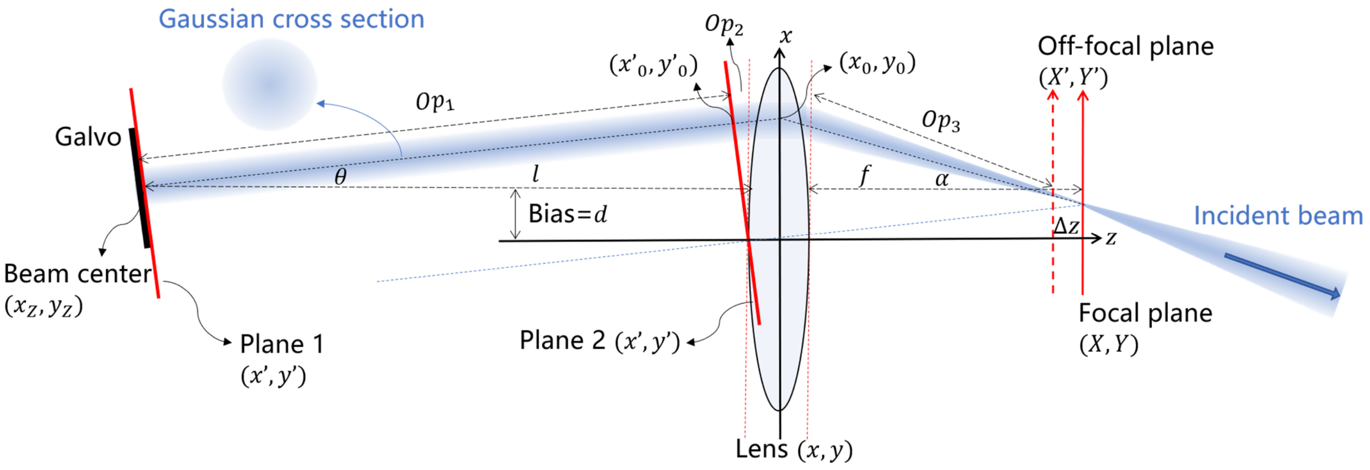


**Figure S1.** The model of the scanning system. Planes 1 and 2 are perpendicular to the beam propagation direction. The coordinate systems of the lens plane, the focal plane, and an off-focal plane are respectively $(x, y)$, $(X, Y)$, and $(X', Y')$. The distance from the galvo mirror and the lens is $l$, the distance between the lens and off-focal plane is $z$, and the focal length is $f$. $\Delta z$ is the difference between $z$ and $f$. The distance bias from the galvo center to the lens optical axis is $d$. The scanning angle is $\theta$ and the incident angle is $\alpha$. $(x_z, y_z)$, $(x'_0, y'_0)$, and $(x_0, y_0)$ present the beam center positions in different planes. Plane 1 is set as the zero reference for optical pathlength.

The beam profile around the focal plane is calculated with the model shown in Fig.S1. The Gaussian beam in Plane 1 is expressed as $U_{01} = A_0\exp\{-[(x' - x_Z)^2 + (y' - y_Z)^2]/r_B^2\}$, where $A_0$ is the amplitude and $r_B$ is the beam radius. The beam is $U_{02} = \exp(ik \cdot Op_1) \cdot A_0\exp\{-[(x' - x'_0)^2 + (y' - y'_0)^2]/r_B^2\}$ in Plane 2. $k$ is the wave number, and $Op_1$ is the optical pathlength between two planes with the value of $[l/\cos\theta - (d + l\tan\theta)\sin\theta]$. The light distribution on the plane before the lens is $U_1 = U_{02} \cdot \exp(ik \cdot Op_2)$, with the approximation of $|U_1| \approx |U_{02}|$, and $Op_2$ equals to $\sin\theta \cdot x$. After the lens modulation of $Op_L = [-(x^2 + y^2)/2f]$, the beam is described as $U_2 = U_1 \cdot \exp(ik \cdot Op_L)$. According to Fresnel diffraction, the light distribution $U_3$ in an off-focal plane is calculated by

$$U_3(X', Y', z) = \iint \frac{1}{i\lambda z} U_2(x, y) \exp\left(i\frac{2\pi}{\lambda} Op_3\right) dxdy \tag{S1.1}$$

, where $Op_3$ is given by $[(X' - x)^2 + (Y' - y)^2 + z^2]^{0.5} \approx z[1 + (X' - x)^2/2z^2 + (Y' - y)^2/2z^2]$. We set the spatial frequencies as $f_x = X'/\lambda z$ and $f_y = Y'/\lambda z$, where $\lambda$ represents wavelength. Based on Fourier optics, the equation (S1.1) can be rewritten as

$$U_3\left(X',Y',z\right) \approx \frac{1}{i\lambda z}\exp\left[ik\left(Op_1+z+\frac{X'^2+Y'^2}{2z}\right)\right]\exp\left(-\frac{x_0^2+y_0^2}{r_B^2}\right)FT_x\left[\exp\left(Ax^2+B_x x\right)\exp\left(ik\sin\theta x\right)\right]\times$$
$$FT_y\left[\exp\left(Ay^2+B_y y\right)\right]=\frac{1}{i\lambda z}\exp\left[ik\left(Op_1+z+\frac{X'^2+Y'^2}{2z}\right)\right]\exp\left(-\frac{x_0^2+y_0^2}{r_B^2}\right)\frac{-\pi}{A}\exp\left[\frac{\left(\pi f_{x'}+iB_x/2\right)^2}{A}\right]\times \tag{S1.2}$$
$$\exp\left[\frac{\left(\pi f_y+iB_y/2\right)^2}{A}\right]$$

, where $A$ stands for $(ik/2C-1/r_B^2)$, $C$ is defined as $zf/(f-z)$, $B_x$ and $B_y$ are respectively $x_0/r_B^2$ and $y_0/r_B^2$, $FT_x$ and $FT_y$ mean one-dimensional Fourier transform, and $f_{x'}$ equals to $(f_x - sin\theta/\lambda)$.
In our study, the scanning direction is along the $x$ −axis. Thus, the beam center is at the position of $(x_0, y_0) = (d + l\tan\theta, 0)$ in the lens plane. For one B-scan, we care about the beam profile on the $X'z$ plane, making $Y'$ equal to zero. Thus, the beam profile is simplified as below.

$$U_3\left(X',Y'=0,z\right)=\left(\frac{1}{i\lambda z}\frac{-\pi}{A}\right)\exp\left(-\frac{x_0^2}{r_B^2}\right)\exp\left[i\frac{2\pi}{\lambda}\left(Op_1+z+\frac{X'^2}{2z}\right)\right]\exp\left[\frac{\left(\pi f_{x'}+iB_x/2\right)^2}{A}\right] \tag{S1.3}$$

This expression is the product of four factors. The vertical amplitude distribution is determined by the first factor. The third factor is largely responsible for the vertical phase distribution. The horizontal distribution is mainly described by the last factor, including beam divergence, horizontal propagation, etc. In the coordinate system of the lens, the light distribution function is expressed as

$$U_L\left(x,z,\theta,d\right)=A\left(x,z,\theta,d\right)\exp\left[ik\cdot r\left(x,z,\theta,d\right)\right] \tag{S1.4}$$

, where $x = X'$, $A = \text{abs}(U_3)$, $r = \text{phase}(U_3)/k$, and the beam center position $x_0$ is the function of $d$ and $\theta$. Since the signal intensity of a scatterer at the depth $z_0$ is approximatively proportional to the illumination amplitude $A(x, z_0)$, the image intensity can be corrected along vertical direction by multiplying $1/\max[A(x, z_0)]$. It must be pointed out that, strictly speaking, the light profile described by equation (S1.3) is only valid for the scatterers located at $Y' = 0$. Scatterers within $Y' \in [-R(z), 0) \cup (0, -R(z)]$, where the beam radius $R$ is a function of depth $z$, will be exposed to a different profile and will be present in the image data.

## S2: Image registration model

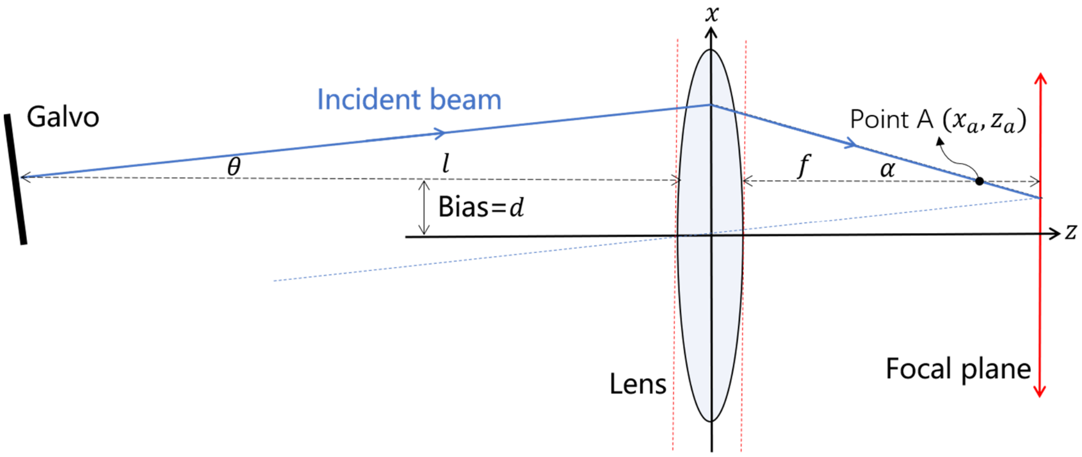


**Figure S2.** The geometrical optical model of the scanning system. $l$ is the distance from galvo and the lens, $f$ is the focal length, $d$ is the distance bias, $\alpha$ is the incident angle, and $\theta$ is the scanning angle. Point A $(x_a, z_a)$ is chosen for analysis. Galvo center is the reference zero for optical pathlength.

The relationship between the pixels in the OCT image and the points in real space is established for image registration. In the OCT image, the horizontal coordinate is on behalf of the scanning angle and the vertical coordinate is the

optical pathlength. Equation (S2.1) is derived from the geometrical optical model in Fig.S2, where $(x_a, z_a)$ is the spatial position of Point A, $\theta$ is the scanning angle at which the beam centerline can pass through Point A, and $Op$ is the one-way optical pathlength. Incident angle $\alpha$ is modulated by distance bias $d$, as shown in equation (S2.2). With a refractive index of $N$, the incident angle changes to $\arcsin(\sin\alpha/N)$. For a point $(x_a, f)$ in the focal plane, its scanning angle $\arctan(x_a/f)$ is independent of $d$. Scanning a flat surface in the focal plane will generate a parabolic curve with a quadratic coefficient of $(f-l)\theta^2/2$, which can measure $l$. If the scanning angle is zero, the beam is supposed to go through the lens focus. The optical pathlength from galvo to the focus approximates $(l+f)$, meaning that the focus position is basically constant in OCT images taken at different angles.

$$\begin{cases} x_a = f_1(\theta, d) = (l\tan\theta + d) - z_a \tan\alpha \\ z_a = f_2(\theta, d, Op) = \left[ Op - \dfrac{l}{\cos\theta} + \dfrac{(l\tan\theta + d)^2}{2f} \right] \cos\alpha \end{cases} \tag{S2.1}$$

$$\alpha = \arctan \frac{(l\tan\theta + d) - f\tan\theta}{f} \tag{S2.2}$$

The pixel coordinate in OCT image is $(u, v)$. The horizontal coordinate $u$ is considered as $\tan\theta \cdot f$ and the vertical coordinate $v$ is the optical pathlength to the reference arm. To calculate the spatial locations of the OCT pixels, the difference between $v$ and $Op$ must be calculated. Since the pixel corresponding to the focus is fixed at the location $(u_f,\ v_f)$, another pixel's $Op$ can be calculated as $[(v - v_f) + (l+f)]$ and the scanning angle error is estimated by $\arctan(u_f/f)$. Thus, the relationship of $(x, y)$ and $(u, v)$ is written below.

$$\begin{cases} x = f_x(u, v, d, u_f, v_f) = f_1\left( \arctan\dfrac{u}{f} - \arctan\dfrac{u_f}{f}, d \right) \\ z = f_z(u, v, d, u_f, v_f) = f_2\left( \arctan\dfrac{u}{f} - \arctan\dfrac{u_f}{f}, d, v - v_f + l + f \right) \end{cases} \tag{S2.3}$$

Different angular images can be fused together by this equation. For imaging registration, roughly locating $(u_f,\ v_f)$ is necessary, the estimated position of which can be chosen as the pixel that has nearly equal distances to the sample surfaces in all the different angular images or the smallest possible average distance. Sample surfaces should be recognized, and the optical pathlength in the sample is the product of the geometric length of the refractive index.

**S3: Point spread function**

The ideal backscattered signal of a scatterer is determined by the beam profile. According to the beam function in equation (S1.4), if the scanning angle $\theta$ varies from $\theta_1$ to $\theta_2$, the signal $S_A$ of the scatterer at $(x_a, z_a)$ is given by equation (S3.1). The phase is double that of the beam's reverse phase, because the optical pathlength is twice that of the incident beam. The scatterer's pixels $(u_a,\ v_a)$ in the OCT image are expressed as equation (S3.2) according to the model in supplementary S2. The amplitude of $S_A$ achieves the maximum when the beam centerline exactly passes through the scatterer, and the corresponding scanning angle $\theta_{max}$ is solved by equation (S2.1) ($\theta_{max}$ is proved to be the same as the angle making $|U_L|$ maximum in equation (S1.4)). Demonstrated by equation (S3.3), the amplitude is a Gaussian function of $u$ and reaches the maximum at $u_a(\theta_{max})$. The scatterer image diameter is the same as the beam size at the depth of $z_a$. It implies that PSF can be derived from the beam function.

$$S_A(x_a, z_a, [\theta_1, \theta_2], d) \propto A(x = x_a, z = z_a, \theta = [\theta_1, \theta_2], d) \exp\left[ ik \cdot 2r(x = x_a, z = z_a, \theta = [\theta_1, \theta_2], d) \right] \tag{S3.1}$$

$$\begin{cases} u_a = \tan([\theta_1, \theta_2]) \cdot f \approx [\theta_1, \theta_2] \cdot f \\ v_a = r(x_a, z_a, [\theta_1, \theta_2], d) + v_f - (l+f) \end{cases} \tag{S3.2}$$

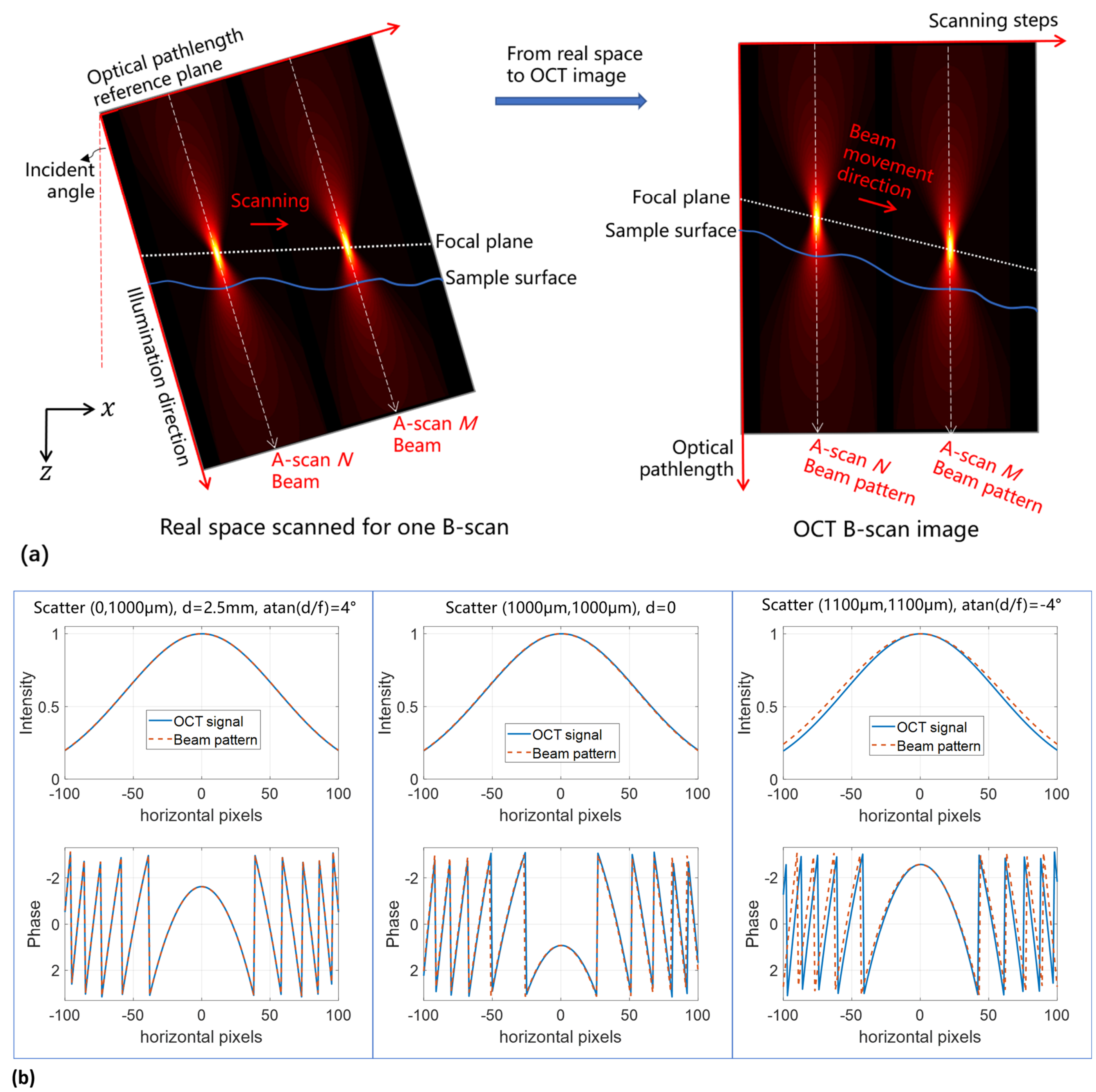


**Figure S3.** The relationship between the scatterer signal and the beam distribution. (a) For one B-scan with angular illumination, the real space scanned by the beam is not a rectangle but close to a rhomboid. In angular OCT images, the sample is rotated by the incident angle and the beam pattern is along the vertical direction, while the focal plane image is oblique. (b) The simulated signals of three scatterers and the wavefronts of beam patterns. The OCT phases in the images are reversed and halved. Tiny errors appear when the distance bias $d$ is not zero or the scatterer is far away from the focus in the horizontal direction. The focus position is at (0,0), the focal length is 36 mm, distance from galvo to the lens is 59 mm, beam diameter is 5.4 mm (1/e$^2$), and the wavelength is 920 nm.

$$|S_A| \propto |U_L| \propto \exp\left[\frac{-\pi^2\left(\frac{u}{\lambda f}-\frac{x_a}{\lambda z_a}\right)^2-\frac{\pi x_0 k}{z_a f}\left(f-z_a\right)\left(\frac{u}{\lambda f}-\frac{x_a}{\lambda z_a}\right)+\frac{x_0^2}{r_B^4}}{\frac{r_B^2 k^2\left(f-z_a\right)^2}{4 z_a^2 f^2}+\frac{1}{r_B^2}}\right] \tag{S3.3}$$

$$PSF\left(u,v,\theta,d{=}0\right)=A\left(u,v,\theta,0\right)\exp\left[ik\cdot 2r\left(u,v,\theta,0\right)\right] \tag{S3.4}$$

Although the real beam is artificially inclined by the distance bias $d$, in the OCT images the corresponding beam pattern $U_B$ is always vertical and moves along the focal plane during scanning, as shown in Fig.S3 (a). $U_B$ can be expressed as $U_L(u, v, \theta, d = 0)$, whose focus has a dynamic vertical coordinate of $v_a(x_a = f_1(\theta, d), z_a = f, \theta, d)$ according to equation (S2.1) and equation (S3.2). The coordinates $P_S$ of the scatterer in the OCT image are $\left(u_a(\theta_{max}), v_a(\theta_{max})\right)$, and the scattering signal is proportional to the convolution of $abs(U_L) \cdot exp[2Phase(U_L)]$ and $P_S$. Thus, the PSF is expressed by equation (S3.4). Fig.S3 (b) presents the simulated signals based on the above theory.

**S4: Comparison of two image registration methods**

The 'rotation + translation' image registration is a common method for angular compounding [28]. It can be expressed as equation (S4.1), where $(u_c,\ v_c)$ is the rotation center and $\varphi$ is the rotation angle. The agar-bead phantom in Fig.2 is processed by this 'rotation + translation' method, as illustrated in Fig.S4(a, c), here $(u_c,\ v_c)$ is the same as the focus position $(u_f,\ v_f)$ and $\varphi$ equals to the incident angle $\arctan(d/f)/N$ ($N$ is refractive index). The registered image shows that the angular images are fused well in the horizontal central region, but the mismatches increase with the distance from the horizontal center. These mismatches can be prevented by our proposed method, as demonstrated in Fig.S4(b, d).

Our geometrically-motivated method, equation (S2.1), can be written as equation (S4.2) using the approximations $\sqrt{1 + \left[(u - u_f)/f\right]^2} \approx 1 + 0.5\left[(u - u_f)/f\right]^2$ and $tan\left[arc\tan(u/f) - \arctan(u_f/f)\right] \approx (u - u_f)/f$, which is a similar expression to equation (S4.1). The three main differences between the proposed model and the 'rotation + translation' model are image scaling, the quadratic mapping between $(x, z)$ and $(u, v)$, and the rotation angle (the rotation angle $\varphi$ in equation (S4.1) is a constant value while the α in equation (S4.2) is a function of $(u - u_f)$). For a small scanning range, the incident angle $\alpha$ approximates to $\arctan(d/f)$, simplifying equation (S4.2) to be equation (S4.3). Usually, the galvanometer mirror is located in the back focal of the lens, implying $l = f$ and $\alpha = \arctan(d/f)$, and the model can be further reduced to equation (S4.4), where the quadratic deformation is removed. In cases where it is hard to guarantee that $l = f$, for example, in a system where the distance from the galvanometer mirror to the lens is fixed but a different focal-length lens is used, the simple 'rotation + translation' operation cannot achieve a good registered image except in the horizontal central region. In summary, the proposed method is more general.

$$\begin{bmatrix} x \\ z \end{bmatrix} = \begin{bmatrix} \cos\varphi & -\sin\varphi \\ \sin\varphi & \cos\varphi \end{bmatrix} \begin{bmatrix} u - u_f \\ v - v_f \end{bmatrix} \rightarrow \begin{cases} x = (u - u_f)\cos\varphi - (v - v_f)\sin\varphi \\ z = (u - u_f)\sin\varphi + (v - v_f)\cos\varphi \end{cases} \tag{S4.1}$$

$$\begin{cases} x \approx \dfrac{l}{f}(u - u_f)\left(1 - \dfrac{d}{f}\sin\alpha\right) - (v - v_f + f)\sin\alpha + \dfrac{l^2 (u - u_f)^2}{f^2}\dfrac{(f - l)}{2lf}\sin\alpha - \dfrac{d^2}{2f}\sin\alpha + d \\ z \approx \dfrac{l}{f}(u - u_f)\dfrac{d}{f}\cos\alpha + (v - v_f + f)\cos\alpha - \dfrac{l^2 (u - u_f)^2}{f^2}\dfrac{(f - l)}{2lf}\cos\alpha + \dfrac{d^2}{2f}\cos\alpha \end{cases} \tag{S4.2}$$

$$\begin{cases} x \approx \dfrac{l}{f}(u - u_f) - (v - v_f + f)\sin\alpha + \dfrac{l^2 (u - u_f)^2}{f^2}\dfrac{(f - l)}{2lf}\sin\alpha + d \\ z \approx \dfrac{l}{f}(u - u_f)\sin\alpha + (v - v_f + f)\cos\alpha - \dfrac{l^2 (u - u_f)^2}{f^2}\dfrac{(f - l)}{2lf}\cos\alpha + \dfrac{d}{2}\sin\alpha \end{cases} \tag{S4.3}$$

$$\begin{cases} x \approx (u - u_f) - (v - v_f + f)\sin\alpha + d \\ z \approx (u - u_f)\sin\alpha + (v - v_f + f)\cos\alpha + \dfrac{d}{2}\sin\alpha \end{cases} \tag{S4.4}$$

In practice, the equations (S4.2~S4.4) are easier to use compared with the original expression (S2.1). The incident angle is calculated by equation (S2.2) and the distance between the gavlo and lens is given by measuring the quadratic

coefficient $(f-l)\theta^2/2$ of the image of a flat plate placed in the focal plane with vertical illumination ($d=0$), as shown in Fig.S4(e). Another practical method is to directly measure the vertical deformation of the flat surface in the focal plane with the vertical illumination. According to the above equations, the vertical deformation is the quadratic term, $-\left[l\left(u-u_f\right)/f\right]^2[(f-l)/2lf]$, which is independent of the vertical coordinate and can be used for other angular images. After removing the quadratic deformations, an angular image can be mapped to the real space by the 'rotation + translation' operations. The quadratic deformation can be eliminated by applying a 4f telescope in the sample arm to place the scanning galvo mirror at the back focal of the objective, but it needs more optical calibrations and corresponding changes in the reference arm, e.g. dispersion compensation.

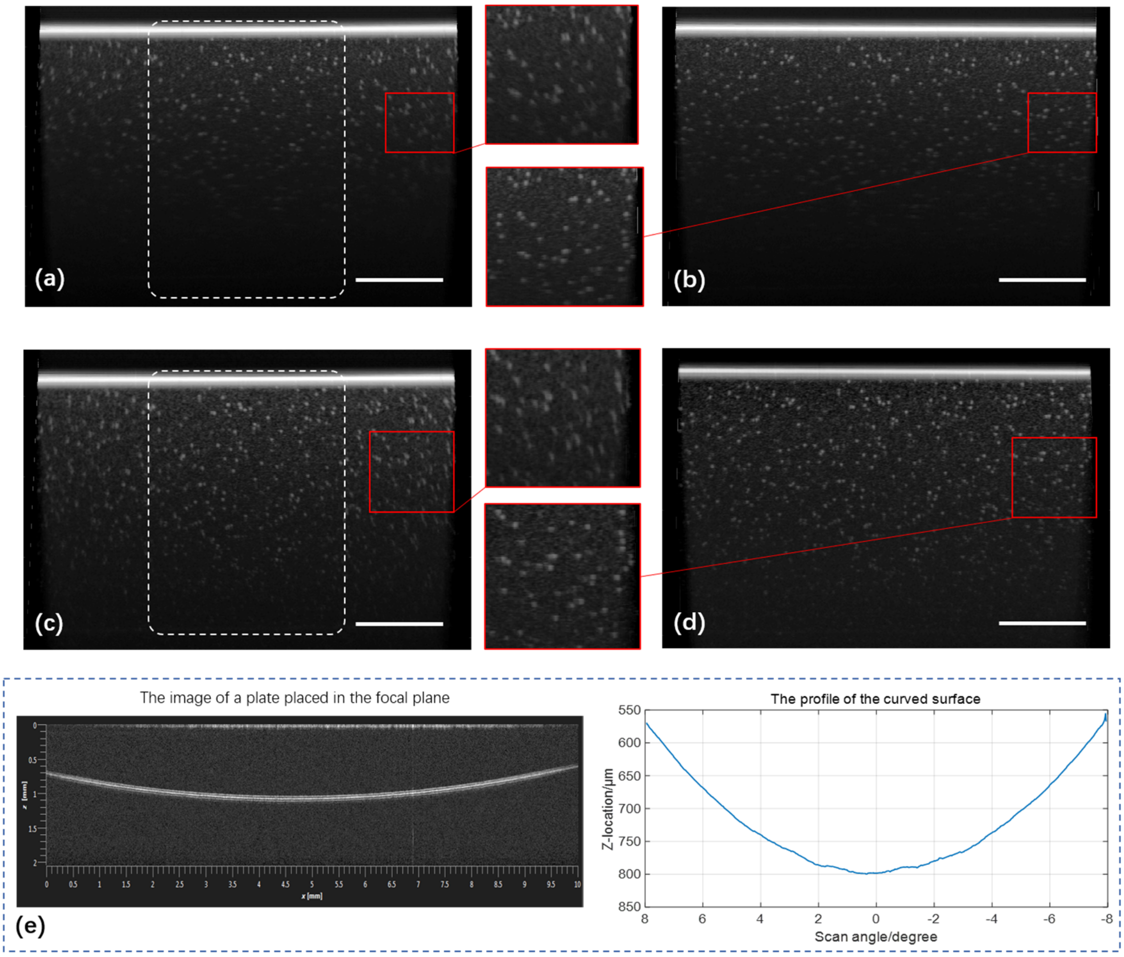


**Figure S4.** (a) The registered image with 'rotation + translation', (b) The registered image with the proposed method, (c) The digitally-focused and registered image with 'rotation + translation', (d) The digitally-focused and registered image with the proposed method. (a, b) In the horizontal central region (marked by the white rectangles), the image registration performs well. In the region away from the horizontal center, apparent mismatches can be found (such as beads in the red rectangles). (b, d) is the same as Fig.2(b, c) (e) Scan a flat plate placed in the focal plane and recognize its surface profile. Scale bar, 200 μm in (a-d).

### S5: Digital focusing with unity magnitude

Fig.S5(a) and (b) use simulations to compare the matched filter, $MF=A(u,v,\theta,0)\exp[-2k\cdot r(u,v,\theta,0)]$, and its unity magnitude form (uniform amplitude in frequency domain), $MF_U=FT_u^{-1}\{exp[i\cdot Phase(FT_u(MF))]\}$, where $FT_u$ is the Fourier transform along horizontal direction $u$, $FT_u^{-1}$ is the inverse transform, and $Phase$ function returns the phase angles. Ideally, if all the information of the $x$-$y$ plane at a given depth is used for digital focusing, the refocused image intensity should be the same with respect to the depth since the luminous flux is constant along the light propagation. For a give depth, our method only utilizes the signals in $x$-direction, resulting in the intensity difference

in $z$-direction. Thus, $MF$ is multiplied by $1/\max_v(A)$ to normalize the vertical intensity, where $\max_v(A)$ means the maximum value at the depth $v$. For $MF_U$ filter, we found that $1/[\max_v(A)]^{0.5}$ is the proper normalization factor, and the digital focusing can be processed by $FT_u^{-1}\{exp[i \cdot Phase(FT_u(MF))] \cdot FT_u(OCT/[\max_v(A)]^{0.5})\}$. In Fig.S5(a) and (b), the diameters of the two refocused beams are constant at all depths. The size of the focused beam by $MF_U$ is the same as the focal size and 27 % smaller than that by $MF$, as shown in Fig.S5(b), because $MF_U$ can fully use all the spatial frequencies. $MF_U$ introduces sidelobes into the defocusing area of the refocused image, which may adversely impact the contrast and penetration depth. We did try $MF_U$ for real data in Fig.S5(c). The contrast of the image processed by $MF_U$ gets worse with increasing the out-of-focus distance. For example, the beads in the defocusing region marked by white rectangle are barely visible, which are much more recognizable in the image given by $MF$. Fig.S5(d) shows the specific profiles of the six selected beads. The bead diameter in the $MF_U$ image is 9~13 % smaller than that in the $MF$ image, while the reduction in contrast can be as large as 60~80 %. Briefly, although $MF_U$ filter generates a little better resolution, $MF$ filter contributes to a higher contrast and a deeper penetration.

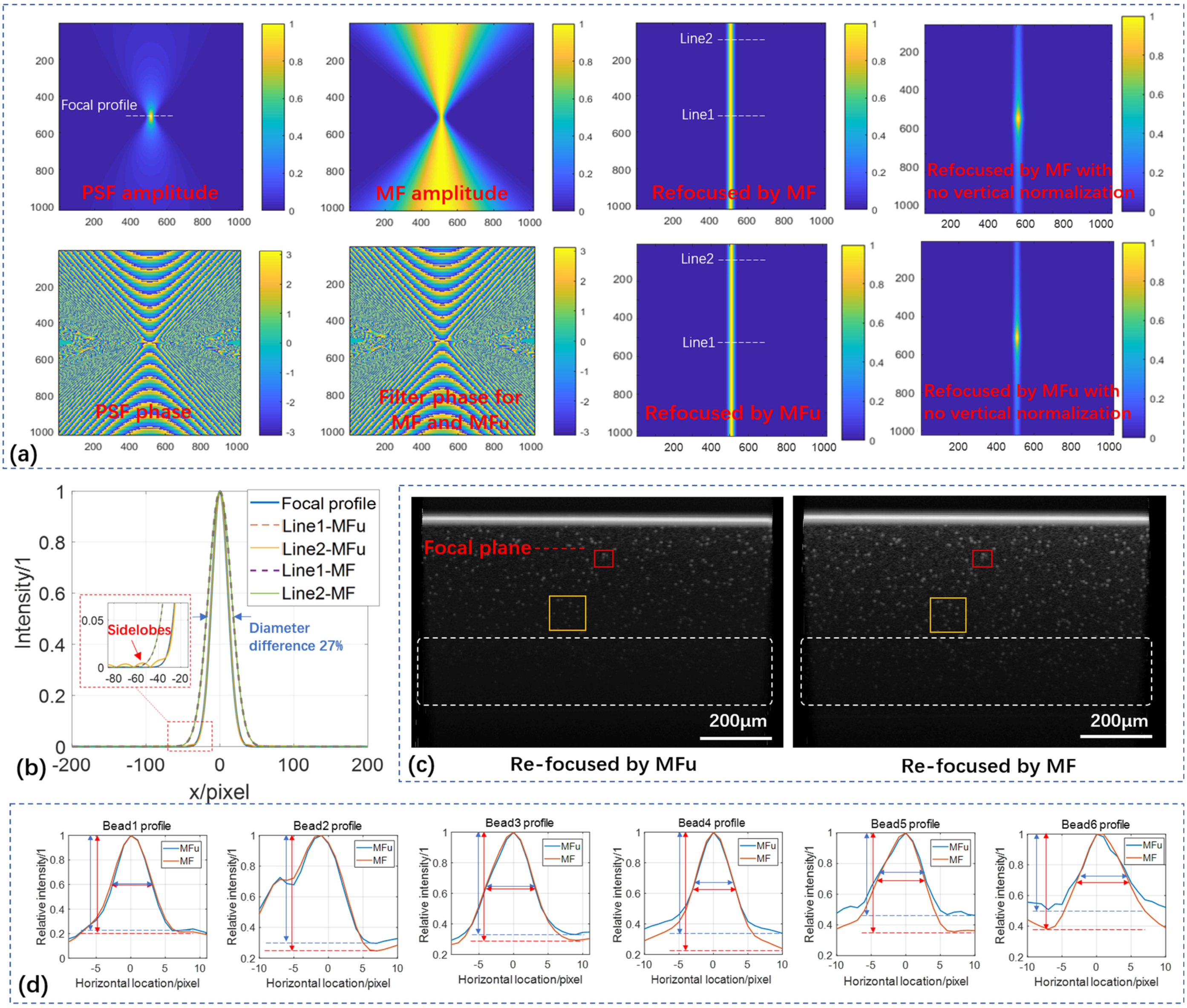


**Figure S5.** (a) Simulations to compare $MF$ and $MF_U$. (b) The profiles of the focused beams along Line 1 and Line 2. (c) Digital focusing of the real data in Fig.2. Beads 1~3 in the red rectangles are close to the focal plane and Beads 4~6 in the yellow rectangles are away from the focal plane, and their exact locations are pointed out in Fig.2(g) and (h). (d) The intensity profiles of the beads. For Beads 1~3, the contrasts (the ratio of signal to background) in the $MF$ image are 8~28 % higher than those in the $MF_U$ image, but the diameters (FWHM) in the $MF$ image are 9 % larger. For Beads 4~6, the contrasts and the diameters in the $MF$ image are 60~80 % higher and 13% larger, respectively.

### S6: Multiple-detection design

Based on the setup in reference [43], a design containing no moveable parts is proposed for fast imaging in Fig.S6 to explore the practicability of our implementation in a clinical/industrial environment. A swept source is used because it can provide OCT with a high-speed scanning using balanced photodetectors, e.g. avalanche photodiode (APD) array or photomultiplier (PMT) array, instead of a line-camera in the spectrometer. The collimated and expanded Gaussian beam from the single mode fiber (SMF) passes through the aperture to generate a more uniform illumination. The incident lights illuminates the sample from different angles, from $\alpha_1$ to $\alpha_N$. The back-scattered light with respect to a specific incident angle is spatially coded and incident on a certain pixel of the array detector. Thus, the intensity distribution across the detector array records all the angular signals concurrently. A-scans are acquired in the same way as for swept-source OCT (SS-OCT). Following the process flow diagram in Fig.S6, the raw data can be converted into the digitally-focused and speckle-reduced image in real time.

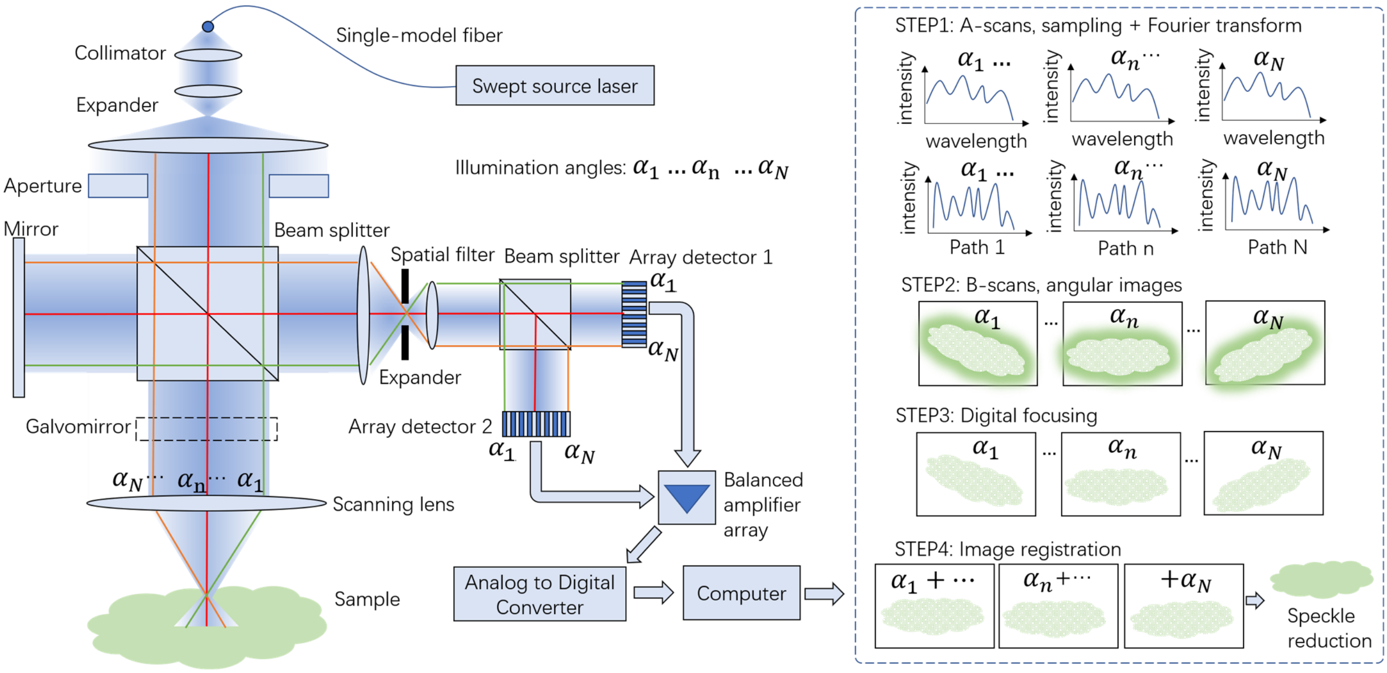


**Figure S6.** The multiple-detection design for fast imaging, hardware setup (left) and data process flow (right).
